# Use of single guided Cas9 nickase to facilitate precise and efficient genome editing in human iPSCs

**DOI:** 10.1038/s41598-021-89312-2

**Published:** 2021-05-10

**Authors:** Pan P. Li, Russell L. Margolis

**Affiliations:** 1 Department of Psychiatry and Behavioral Sciences, Division of Neurobiology, Johns Hopkins University School of Medicine, CMSC 8-121, 600 N. Wolfe St, Baltimore, MD 21287 USA; 2 Department of Neurology, Johns Hopkins University School of Medicine, Baltimore, MD USA

**Keywords:** Neuroscience, Stem cells

## Abstract

Cas9 nucleases permit rapid and efficient generation of gene-edited cell lines. However, in typical protocols, mutations are intentionally introduced into the donor template to avoid the cleavage of donor template or re-cleavage of the successfully edited allele, compromising the fidelity of the isogenic lines generated. In addition, the double-stranded breaks (DSBs) used for editing can introduce undesirable “on-target” indels within the second allele of successfully modified cells via non-homologous end joining (NHEJ). To address these problems, we present an optimized protocol for precise genome editing in human iPSCs that employs (1) single guided Cas9 nickase to generate single-stranded breaks (SSBs), (2) transient overexpression of BCL-XL to enhance survival post electroporation, and (3) the PiggyBac transposon system for seamless removal of dual selection markers. We have used this method to modify the length of the CAG repeat contained in exon 7 of *PPP2R2B*. When longer than 43 triplets, this repeat causes the neurodegenerative disorder spinocerebellar ataxia type 12 (SCA12); our goal was to seamlessly introduce the SCA12 mutation into a human control iPSC line. With our protocol, ~ 15% of iPSC clones selected had the desired gene editing without “on target” indels or off-target changes, and without the deliberate introduction of mutations via the donor template. This method will allow for the precise and efficient editing of human iPSCs for disease modeling and other purposes.

## Introduction

Genome editing of human iPSCs has been widely used for modeling human diseases. Current CRISPR/Cas9 genome editing approaches in human pluripotent stem cells (hPSCs) rely on the introduction of double-stranded breaks (DSBs), which can result in insertion or deletion (indels) of DNA sequences, genome instability, chromosomal translocations, apoptosis, and acquisition of potentially oncogenic mutations^1–3^.

Most genetic variants that contribute to disease arise from specific insertions, deletions, or base substitutions that require precise editing methods to correct or model. Homology-directed repair (HDR) stimulated by DSBs has been widely used to install precise DNA changes. Apart from DSBs generated by WT Cas9, double nicking (DN) by Cas9 D10A nickase (Cas9n) and paired off-set guide RNAs (gRNAs) also generates DSBs^4^, but with significantly less off-target activities^5^. The efficiency of HDR following CRISPR/Cas9-induced DSB is rather low, compared with the efficiency of non-homologous end joining (NHEJ)^6,7^. Moreover, even in cells where HDR has successfully taken place, lingering Cas9 and gRNAs can generate DSBs and re-introduce undesirable “on target” indels to the edited allele, as well as to the second allele that has not undergone HDR^8,9^. To avoid indels on the already edited alleles post HDR, “blocking” mutations at the gRNA or PAM sequence are often intentionally introduced to the donor at the gRNA or PAM sequence^10,11^. However, this method introduces mutations in addition to the desired changes, compromising the fidelity of the newly generated iPSC lines. In addition, such “blocking” mutations do not prevent the generation of indels on the second allele.

We therefore explored the use of HDRs induced by single-stranded breaks (SSBs) in human iPSCs. It was previously shown that SSBs induced by single guided Cas9n are also repaired by HDRs, but the efficiency of SSB-induced HDRs is much lower than DSB-induced HDR^6^. Notably, single nicking (SN)-induced HDRs were detectable in HEK293T cells but not in human iPSCs^5^.

Here, we report an optimized genome editing protocol for human iPSCs employing SN, transient BCL-XL overexpression^12^, and a donor construct with a PiggyBac transposase removable dual selection cassette. Using the editing of the CAG repeat region in human *PPP2R2B* exon 7 as an example, we demonstrate that SN, but not DN, is associated with indel-free and precise editing outcome, while maintaining ~ 15% targeting efficiency. This method has the potential of significantly improving the precision and efficiency of genome editing in human iPSCs for disease modeling and beyond.

## Results

### BCL-XL overexpression enhances indel formation in human iPSCs treated with Cas9n and paired but not single gRNAs

DN by Cas9n and paired gRNAs generate efficient indels with significantly less off-target activities than wildtype Cas9^5^. Single nicking by Cas9n and a single gRNA produces SSBs, but does not generate indels or induce detectable HDRs in human iPSCs^6^. However, it was recently reported that overexpression of BCL-XL leads to a ∼20- to 100-fold increase in the efficiency of DSB-induced HDR and a ~ fivefold increase in NHEJ at multiple loci in human iPSCs ^12^.

**Figure 1:**
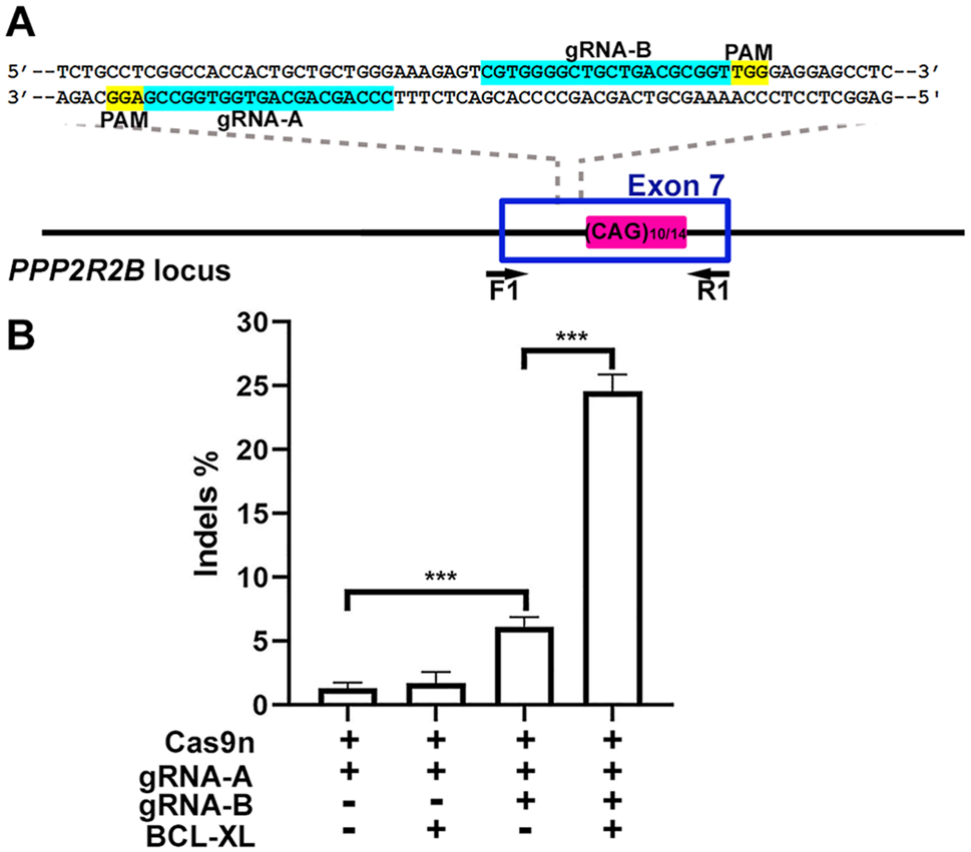
DN, but not SN, promotes indel formation in human iPSCs. (**A**) Schematic representation of two gRNA sequences (on two opposite strands) upstream of the CAG repeat in exon 7 of human *PPP2R2B* gene. Primers F1 and R1 were used for PCR amplification. (**B**) Indel frequencies calculated by TIDE using sequence traces. BCL-XL overexpression increased indels induced by DN but did not increase indels induced by SN. Mean and SEM. N = 3 independent experiments. One-way ANOVA F(3,8) = 150.0; p < 0.001; Tukey’s multiple comparison test ***p < 0.001.

To test if BCL-XL overexpression boosts indel formation induced by SN, paired gRNAs or a single gRNA (Fig. 1A) along with Cas9n were electroporated into human control iPSCs with or without BCL-XL. 48 h after electroporation, human iPSCs were harvested, and genomic DNAs extracted. F1/R1 primers were used to amplify the region that contains the gRNA sequences and the CAG repeat. The human control iPSC line used for this study had 10 and 14 CAG triplets, respectively, in the two alleles of exon 7 of *PPP2R2B*; this slight difference in length (included in the PCR product by F1/R1 primers) greatly decreased the sensitivity of T7 endonuclease 1 (T7E1) assay for indel detection^13^ (data not shown). As an alternative, TIDE (Tracking of Indels by Decomposition)^14^ was used to analyze the percentage of sequences with indels (Indels%), within each sample, compared to the same region of genomic DNA from iPSCs treated with Cas9n and a control gRNA. As shown in Fig. 1B, DN was more efficient in generating indels than SN, and BCL-XL overexpression further increased indels generated by DN by ~ fivefold, consistent with a previous report^12^. However, BCL-XL did not increase indels generated by SN.

We combined BCL-XL overexpression and SN, as BCL-XL expression has been previously reported to increase hiPSC survival post electroporation^12^, and SN has been reported to protect the edited allele from re-cleavage and the second allele from “on-target” indels^15,16^. We tested this idea by attempting to generate isogenic iPSC lines with different lengths of the CAG repeat in exon 7 of human *PPP2R2B* gene (Fig. 1A), part of our long term goal of generating cell models of spinocerebellar ataxia type 12 (SCA12), a disorder caused by expanded CAG repeats at this locus^17,18^.

### Efficient and precise genome editing in human iPSCs for disease modeling

**Figure 2:**
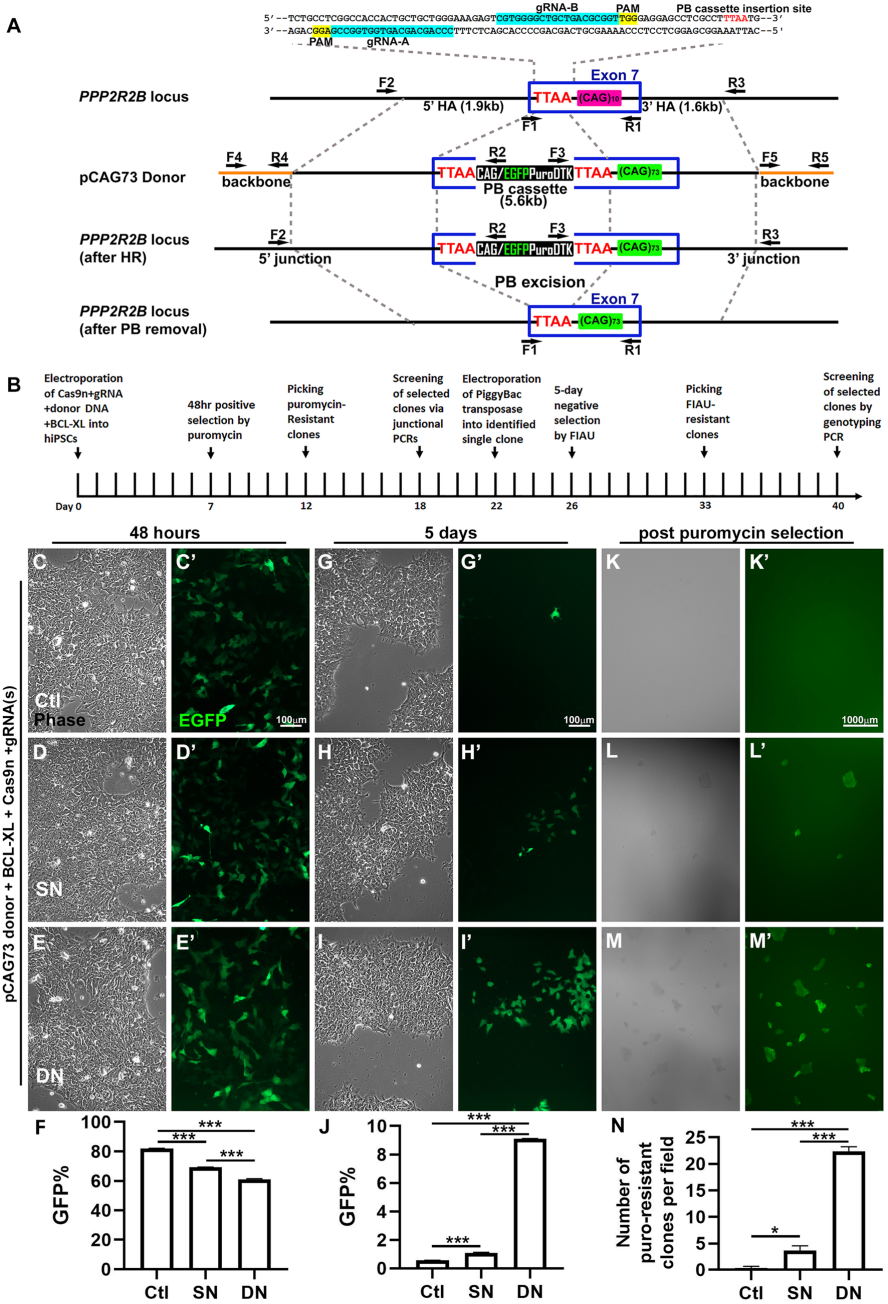
Genome editing at the *PPP2R2B* locus in human iPSCs. (**A**) A schematic diagram (not drawn to scale) showing *PPP2R2B* exon 7, donor construct, PiggyBac (PB) selection cassette, and primer locations. HA: homologous arms; Puro: puromycin N-acetyltransferase gene; DTK: truncated thymidine kinase gene. EGFP/Puro/TDK was driven by the CMV early enhancer/chicken β actin (CAG) promoter. TTAA site was used for PB cassette insertion and removal. (**B**) Timeline of genome editing, including positive/negative selection, screening, excision, and rescreening. (C-N) GFP expression during the process of genome editing. GFP signal from the unincorporated donor is stable for up to five days post electroporation. GFP expression level was similar at 48 h post electroporation across control, SN, and DN conditions (**C**–**F**). EGFP declines rapidly in the control and was only marginally detected by day 5 (**G**–**J**’). Percentage of GFP positive cells in F and J was quantified by flow cytometry. N = 3 different wells of cells per condition. (**F**) One-way ANOVA F(2,6) = 1210; p < 0.001; Tukey’s multiple comparison test; ***p < 0.001. (**J**) One-way ANOVA F(2,6) = 19,852; p < 0.001; Tukey’s multiple comparison test; ***p < 0.001. (**K**–**N**)The number of GFP positive and puromycin resistant clones was substantially greater in cells following DN than following SN, or control treatment. Colony counting was performed at 2X magnification. N = 3 different fields per condition. Mean ± SEM. One-way ANOVA F(2,6) = 253.1; p < 0.001; Tukey’s multiple comparison test. *p < 0.05; ***p < 0.001; ***p < 0.001.

Our goal was to change a normal CAG repeat (10 or 14 triplets) in *PPP2R2B* exon 7 in the human control iPSC line into an expanded CAG repeat with 73 triplets, thereby generating an isogenic human iPSC line for modeling SCA12. Since HDR efficiency in iPSCs is generally low, we introduced selection markers into the donor construct to facilitate detection of cells that have been successfully edited. We chose the PiggyBac (PB) transposon system because it allows seamless removal of the selection markers without altering the endogenous DNA sequence^19,20^. We identified a TTAA site 25 bp upstream of the CAG repeat in exon 7 that could be used as the insertion site for the PB cassette (Fig. 2A). EGFP/Puro/DTK, a fusion of the EGFP gene, the puromycin N-acetyltransferase gene, and the truncated (delta) thymidine kinase gene each separated by T2A peptide sequences was added to the construct for positive selection by puromycin for integration and negative selection by fialuridine (FIAU) for loss of EGFP/Puro/DTK by PB excision^19,21^. The donor construct is shown in Fig. 2A, and the experimental timeline is depicted in Fig. 2B. A 1.9 kb sequence upstream of the TTAA site and a 1.6 kb sequence downstream of the TTAA site were chosen as the 5′ and 3′ homologous arms (HAs), respectively. The 1.6 kb 3′ HA contains 73 CAG triplets to replace the endogenous repeat of 10 or 14 triplets.

We used transient BCL-XL overexpression to boost survival post electroporation^12^, and compared the HDR efficiency of SN and DN. The donor construct, BCL-XL, Cas9n, and the gRNA pair (A and B; DN), a single gRNA (A alone; SN), or a non-targeting control gRNA (Ctl), were electroporated into human control iPSCs. EGFP expression from the donor was monitored in iPSCs post electroporation. At 48 h, DN, SN and control treated cells express EGFP at similar levels (Fig. 2C–E’), as quantified in Fig. 2F. While the EGFP signal declined rapidly in the control treated cells with few EGFP positive cells detectable by day 5 (Fig. 2G and G’), the percentage of EGFP positive cells was much higher in SN or DN treated cells (Fig. 2H-I’), as quantified in Fig. 2J, suggesting that the unincorporated donor plasmid may linger in iPSCs for at least 5 days. After puromycin selection at Day 7, the DN treated cells (Fig. 2M and M’) had 5–10 fold the number of puromycin-resistant clones compared with the SN treated cells (Fig. 2L and L’), while almost no puromycin-resistant clones were detectable after the control treatment (Fig. 2K and K’), as quantified in Fig. 2N. Individual clones were manually picked and expanded in culture.

**Figure 3:**
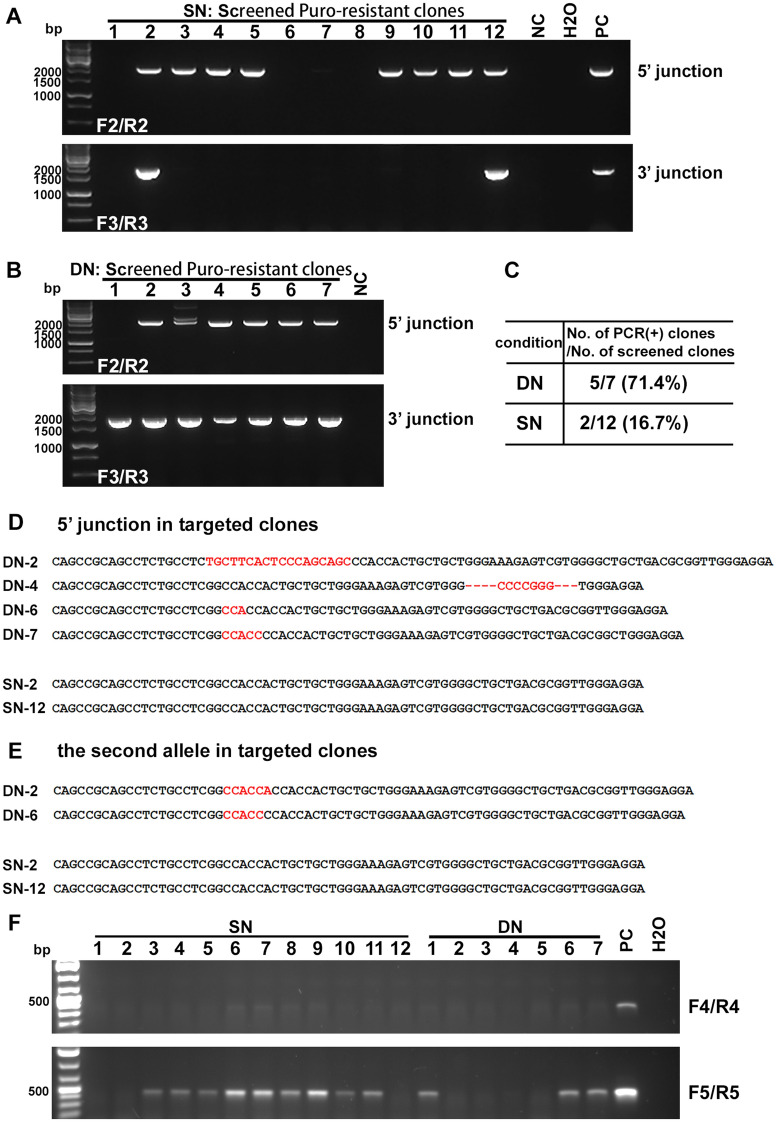
Comparison of DN and SN efficiency and fidelity. (**A**,**B**) PCR-based screening of 5′ and 3′ junctions to identify successfully targeted iPSC clones for SN (**A**) and DN (**B**) treated cells. 1.9 kb band (primers F2/R2) indicates a successfully generated 5′ junction. 1.6 kb band (primers F3/R3) indicate a successfully generated 3′ junction. Primer locations are as indicated in Fig. 2A; unincorporated donor DNA and junctional regions of unsuccessfully edited cells cannot be amplified by these primers. PC: positive control; NC: negative control. (**C**) Summary of targeting efficiency. (**D**) DN, but not SN, is associated with indels in the edited allele. Sequence of the 5′ junction PCR product (primer F2/R2) was used for analysis. The corresponding sequence on the donor was used as the reference for comparison. Indels are in red. (**E**) DN, but not SN, is associated with indels in the second allele that does not undergo HDR. Sequence of PCR product (primer F1/R1) for the second allele was used for analysis. Sequence of the unedited parental line was used as the reference for comparison. Indels are in red. (**F**) Random donor integration in all screened SN and DN clones were examined by PCR using primers F4/R4 (upper image) and F5/R5 (lower image), which bind to the donor plasmid backbone. A diluted donor plasmid was used as the positive control (PC). More integration was detected with F5/R5 primers; SN-2 and -12 clones had no random integration. Primer locations as indicated in Fig. 2A.

PCR using primers F2 and R2, and F3 and R3, was used to screen puromycin-resistant clones for the integrity of 5′ and 3′ junctions, respectively (Fig. 3A,B). The targeting efficiency was 71.4% for DN-treated cells, and 16.7% for SN-treated cells (Fig. 3C).

Next, for each successfully targeted clone, we used Sanger sequencing to examine the integrity of gRNA sequences on both the edited allele (F2/R2) and the allele that did not undergo HDR (F1/R1). While no indels were found in the gRNA region on either allele in any of the targeted clones generated from SN-treatment, all targeted clones generated from DN treatment had indels on both alleles (Fig. 3D,E). The undesirable indels in DN clones rendered them unusable, so we chose to focus on clones generated from SN treatment for further experiments.

To confirm that there were no randomly integrated donors in the positive clones, PCR using primers F4/R4 and F5/R5 was used to screen 12 SN clones and 7 DN clones by amplifying the regions corresponding to the donor vector backbone. PCR results indicate an absence of random donor integration in the positive SN clones 2 and 12, though SN leads to a higher ratio of random donor integration after puromycin selection than does DN (Fig. 3F)**.**

To determine if BCL-XL overexpression promotes HDR induced by SSBs, we compared the targeting efficiency of SN with or without BCL-XL. The donor construct, Cas9n, a single gRNA-A, with or without BCL-XL, were electroporated into human control iPSCs. After puromycin selection at Day 7, individual clones were manually picked and expanded in culture. Junctional PCRs using primers F2/R2 and F3/F3 showed similar targeting efficiency by SN with or without BCL-XL (Fig. S2), indicating that BCL-XL does not in general promote HDR induced by SSBs.

### Removal of PiggyBac selection markers

**Figure 4:**
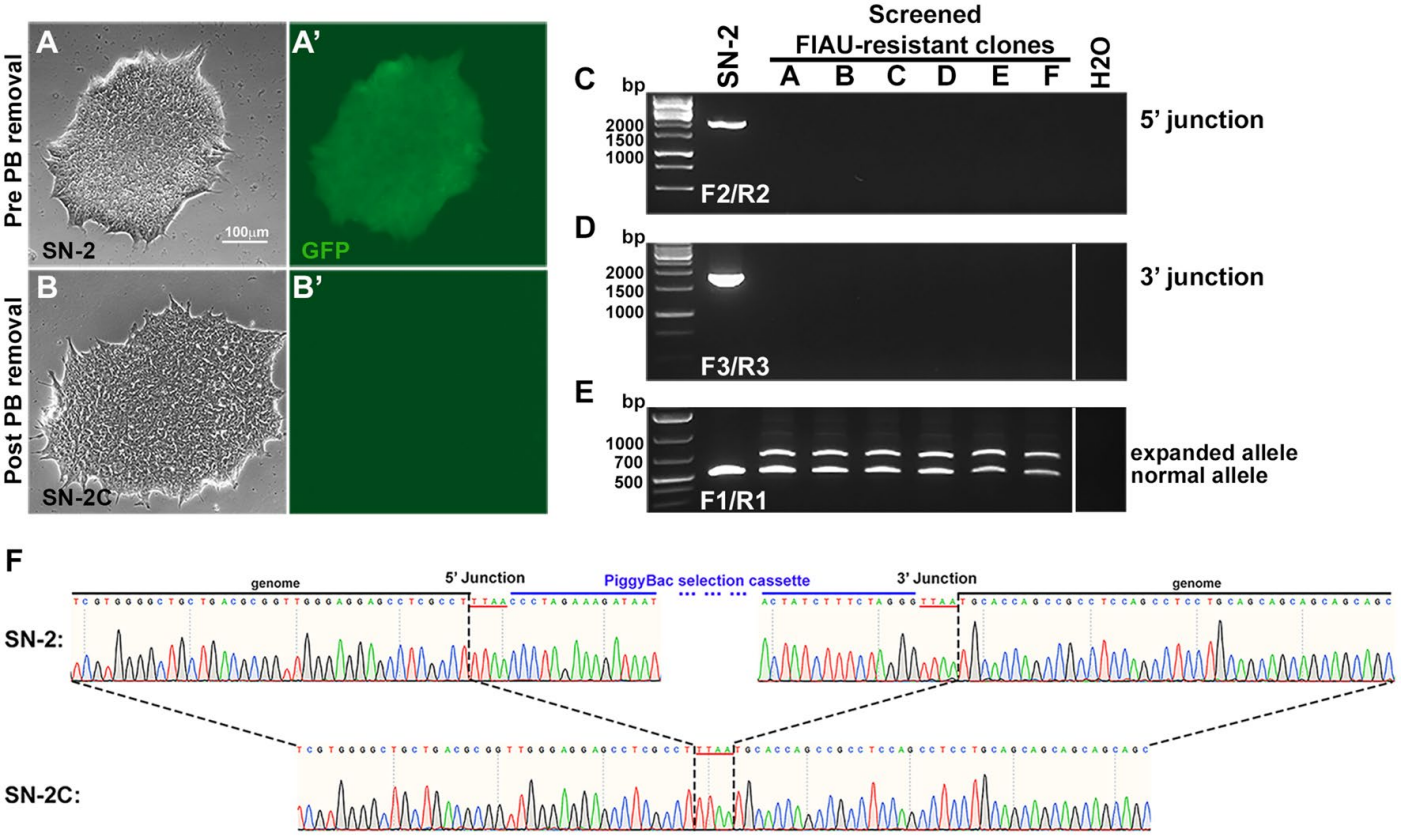
Excision of PiggyBac selection cassette and incorporation of expanded CAG repeat. (**A**–**A**’) GFP from the PB cassette was expressed in clone SN-2 (pre PB cassette removal). (**B**–**B**’) No GFP was detected in the SN-2C clone, indicating successful removal of the PB cassette. (**C**–**D**) PCR confirmed PB cassette removal in targeted iPSC clones that survived the negative election of FIAU. Clone SN-2 (pre PB cassette removal) was used as a positive control. Primers F2/R2 were used to test the 5′ junction and primers F3/R3 were used to test the 3′ junction; absence of band indicates PB-cassette removal. (**E**) PCR (primers F1/R1) confirms the introduction of an expanded CAG repeat of 73 triplets (upper band) into the isogenic iPSC clonal lines A–F (assessed post PB cassette excision). The repeat length of 73 triplets was confirmed by Sanger sequencing. The lower band indicates the presence of the normal length second allele of 14 triplets. Only the unedited normal allele is amplified in the lane with DNA from the SN-2 clone, as the PB cassette is still present in the edited allele of this clone and is too long (5.6 kb) for PCR amplification by primer pair F1/R1. Primer locations are as indicated in Fig. 2A. (**F**) Comparison of Sanger sequencing chromatograms from pre- and post- excision clones confirmed the seamless removal of the PB cassette.

To remove the PB cassette, a plasmid expressing excision-only PB transposase was electroporated into clone SN-2 (Fig. 4A–A’), one of the successfully targeted clones from SN-treated cells (Fig. 3C). Beginning three days post electroporation, a 5-day negative selection was performed using FIAU to select for cells in which the PB cassette was removed. Individual clones that survived FIAU selection were picked, and as expected, did not express EGFP (Fig. 4B–B’). Next, PCRs of junctions were performed to confirm the absence of the PB cassette (Fig. 4C–D). PCR (using primers F1/R1) with subsequent sequencing was performed to verify the successful replacement of a normal CAG repeat (10 CAG triplets) with an expanded CAG repeat (73 CAG triplets) (Fig. 4E). Comparison of sequences of edited alleles from pre- and post- PB cassette removal verified the seamless excision of the PB cassette without changes in the sequences flanking the TTAA site (Fig. 4F). Therefore, the only difference between the parental iPSC line and the final edited SN-2C line is the CAG repeat length of one allele, with no other change in sequence, making the SN-2C line a truly isogenic SCA12 iPSC line.

### Characterization of an isogenic human iPSC clone generated by genome editing

SN does not usually cause off-target indels^5,15,16^. To confirm this finding in our protocol, genomic DNA was extracted from a pool of ~ 50 puromycin-resistant SN-treated clones. Indel% determined by TIDE analysis for the 10 predicted off-target sites ranged from 0.2% to 1.6% (Fig. S1A-B), indicating that single nicking using gRNA-A did not generate off-target indels. Given limitations on the sensitivity of TIDE^14^, we further tested for the presence of indels in the final edited SN-2C line by PCR amplification and Sanger sequencing of each predicted off-target site (Table S1) and no indels were detected (Fig. S3D). The final edited SN-2C line had a normal karyotype (Fig. S3A) and expressed pluripotent markers Sox2 and Oct4 (Fig. S3B-C’’), indicating that the genome editing method did not alter the chromosome stability or pluripotency of the iPSCs.

**Figure 5:**
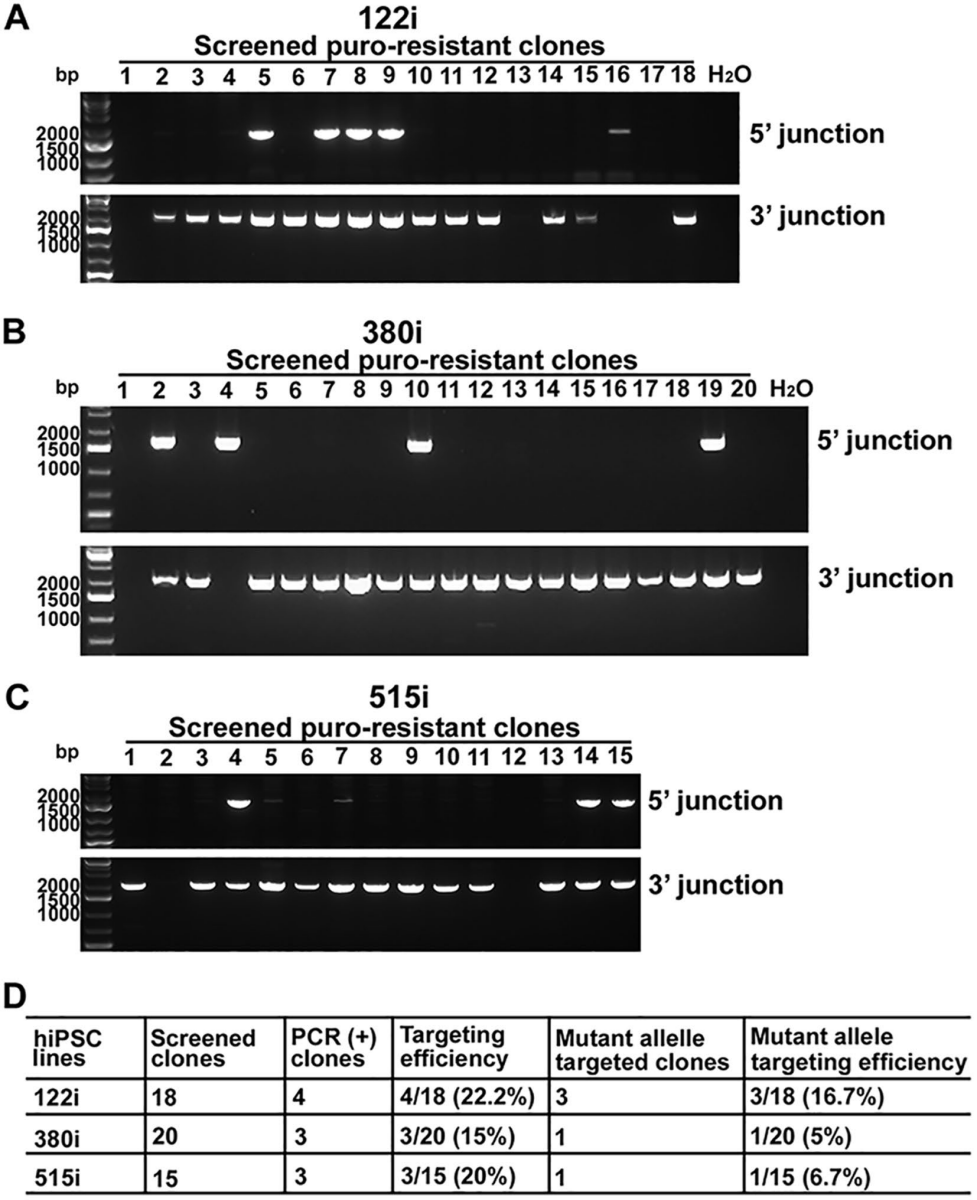
Targeting efficiency of the mutant allele in human SCA12 iPSC lines by SN. (**A**–**C**) Gel images of 5′ and 3′ junctional PCRs in puromycin-resistant clones from three SCA12 (122i, 380i, and 515i) iPSC lines processed with SN and BCL-XL. (**D**). Summary of targeting efficiency in three SCA12 iPSC lines.

As an additional test of our method, we used the same protocol to successfully replace expanded repeats of 55, 65 and 68 triplets in three SCA12 patient-derived iPSC lines (122i, 380i and 515i, Fig. 5) with a normal repeat of 10 CAG triplets, generating isogenic control iPSC lines. Junctional PCR results for puromycin-resistant clones for each SCA12 iPSC lines are shown in Fig. 5A,C. With 15–20 clones screened for each SCA12 iPSC line, the targeting efficiency was 5%-16.67% for the mutant allele (Fig. 5D). The change in repeat length was again accomplished without introduction of mutations at the target locus or off-target indels.

## Discussion

We report that SN, combined with transient BCL-XL overexpression and a removable dual selection cassette, facilitates precise and seamless genome editing of human iPSCs. We applied the method to the CAG repeat of 10 triplets present in exon 7 of *PPP2R2B* of control human iPSCs, generating an isogenic line that contained an expanded CAG repeat with 73 triplets, and to generating isogenic lines with normal repeat lengths from SCA12 patient derived lines with expanded repeats of different lengths. Together, this collection of iPSCs will serve as a useful cell model of SCA12.In previous genome editing using CRISP-Cas9 systems, point mutations were generally deliberately introduced into the gRNA sequences on the donor constructs to prevent the re-cleavage of the edited allele and hence the generation of indels after successful HDR^10,11^. However, such mutations are an additional and unwanted perturbation of the system under investigation, potentially altering transcript expression, splicing, or other gene functions, many of which may not be predictable. Experimental confirmation that these mutations have little or no effect on the system under study may not always be feasible. Our protocol did not require insertion of such mutations into gRNA or PAM sequence, avoiding this complication. With BCL-XL overexpression, DN gave rise to a much higher (~ three-fold) targeting efficiency than SN using the same donor construct (Fig. 3C). However, 100% of the successfully targeted puromycin-resistant clones from the DN-treated cells had indels in the edited allele or the second allele, while no indels were present in any of the successfully targeted puromycin-resistant clones from SN-treated cells (Fig. 3D,E), demonstrating that SN is advantageous over DN in achieving precise editing and preventing indel formation. With the high targeting efficiency, manual selection of < 15 puromycin-resistant clones was sufficient to find successfully edited cells, compared to the > 100 clones typically selected with other genome editing methods, greatly reducing the amount of labor and reagents required for editing iPSCs lines. Seamless alteration of repeat lengths is a particularly challenging application of our protocol. We have so far only applied this method to the precise editing of the *PPP2R2B* locus. While experimental confirmation will be necessary, we anticipate that future studies will demonstrate the applicability of the protocol to editing of other loci and other types of genetic variation.

Use of Cas9n with off-set paired gRNAs leads to reduced off-target activities by 50–1,000 fold compared to WT Cas9^5^. We anticipate that the use of a single gRNA (rather than a pair of gRNAs) together with Cas9n may exhibit even less off-target effects. The use of a single gRNA, rather than a pair of gRNAs, will also halve the amount of effort required to search for potential off-target sites in successfully edited iPSC clones.

Consistent with a recent report^12^, we demonstrated that BCL-XL enhances NHEJ induced by DSBs in human iPSCs (Fig. 1B). It was previously shown that BCL-XL increases HDR induced by DSBs^12^. Here we confirmed that BCL-XL overexpression does not seem to promote HDR induced by SSBs generated by SN (Fig. S2). However, BCL-XL is known to enhance hiPSC survival post electroporation^12^, and therefore BCL-XL is an important component of our protocol. As previously demonstrated^12^, transient or stable BCL-XL overexpression in human iPSCs does not alter the karyotype, indicating the feasibility of its use for genome editing in human iPSCs.

Overall, a protocol combining SN, BCL-XL overexpression and a PiggyBac transposase removable selection cassette provides an efficient and high-fidelity approach to genome editing of iPSCs. This method is applicable to genome editing of iPSCs for a variety of purposes, including modeling other diseases.

## Materials and methods

### Human iPSC (hiPSC) culture

The human control iPSC CS25i-18n2 line was obtained from Induced Pluripotent Stem Cell (iPSC) Core in Cedars-Sinai (Los Angeles, CA). Human SCA12 iPSC lines (122i, 380i and 515i) were generated from SCA12 patient skin fibroblast using an episomal protocol. hiPSCs were grown on Matrigel (Corning, Corning, NY) in Stemflex medium (Thermo Fisher, Waltham, MA) with every day medium change. 0.5 mM EDTA in DPBS was used for regular passaging of iPSCs.

### Plasmid constructs

sgRNA-A (5′-CCCAGCAGCAGTGGTGGCCG-3′) and sgRNA-B (5′-CGTGGGGCTGCTGACGCGGT-3′) sequences were cloned into the pX335-U6-Chimeric_BB-CBh-hSpCas9n(D10A) vector (Addgene #42335^5^), using NEBuilder HiFi cloning kit (NEB, Ipswich, MA). A 1.9 kb 5′ homology arm containing human PPP2R2B intron 6, and a 1.6 kb 3′ homology arm containing human PPP2R2B exon7 (with 73 CAG repeats) and intron 7, were PCR amplified to replace the pre-existing homologous arms in the pJOP-HTT-HR18Q plasmid (Addgene #87228^19^) in order to generate pJOP-PPP2R2B-HR73CAGs plasmid. To enhance the GFP signal in the donor construct, the EF1a short promoter in donor plasmid was replaced by the CMV early enhancer/chicken β actin (CAG) promoter. To achieve that, a 4451 bp Nhe1 (blunted)-Not1 (blunted) fragment from AAT-PB-CG2APtk plasmid (a kind gift from Dr. Tobias Cantz, Addgene #86003^21^) was ligated to a 8231 bp CIP treated AsiSI (blunted)-ClaI (blunted) fragment from pJOP-PPP2R2B-HRCAG73 plasmid using T4 DNA ligase (NEB), checked for correct orientation to obtain the final pCAG73 donor plasmid used for the genome editing.

pEF-BCL-XL plasmid was a kind gift from Dr. Xiao-bing Zhang^12^. The excision only PiggyBac transposase expression vector, which is not propagatable in E. coli, was purchased from System Biosciences (Palo Alto, CA). The excision-only PB transposase^22^ was PCR amplified and cloned into pEMBL-CMV vector (at BamH1 and Not1 sites) using primers PBx-F/PBx-R (Table S1) and NEBuilder HiFi cloning kit (NEB), in order to obtain pEMBL-CMV-PBx plasmid that could be propagated and amplified in E. coli.

### Electroporation of hiPSCs

hiPSCs were dissociated with TryPLE (Thermo Fisher) into single cells, and then washed with DPBS. 2X10^6 cells were electroporated using the Celetrix electroporator, buffer and 120ul pressured tubes (Celetrix, Manassas, VA) with 8–12.5ug total of various plasmids at 630 V for a single pulse of 30 ms^20^. For testing the effect of BCL-XL on indel formation by Cas9 D10A and sgRNAs, 8ug of sgRNA plasmids (8ug for single, or 4ug for each) were used, with or without 4ug of pEF-BCL-XL plasmid DNA. For homologous recombination and positive selection, 5ug sgRNA plasmids (5ug for single, or 2.5ug for each), 5ug pCAG73 Donor plasmid, and 2.5ug pEF-BCL-XL plasmid was used. For removal of piggyBac cassette by negative selection, 10ug of pEMBL-CMV-PBx plasmid was used. After electroporation, the cells were immediately transferred to warm Stemflex medium (Thermo Fisher), and 1X RevitaCell supplement (Thermo Fisher) was added for 18 h to improve survival.

### Selection of hiPSC clones and junction PCRs

Electroporated hiPSCs were passaged at 1:6 ratio using TrypLE whenever the culture reached confluence. Targeted hiPSCs were positively selected by 1 ug/mL puromycin treatment for 48 h from day 7 post electroporation. Surviving colonies were manually picked and expanded for culture and screened by junction PCR using F2/R2 and F3/R3 primers (Table S1). Negative selection for removal of piggyBac cassette harboring selection marker was achieved by 0.25uM fialuridine (FIAU, Sigma, St. Louis, MO) treatment for 5 days starting from Day 3 post electroporation. Surviving colonies were manually picked and expanded for culture and further screened by junction PCRs using F2/R2 and F3/R3 primers.

### Analysis of piggyBac TTAA site post-excision

The genomic region flanking the TTAA site (piggyBac transposase excision site) from pre and post-excision clones were PCR amplified with primers F2/R2 and F1/R1 primers (Table S1), respectively. Amplicons were visualized on 1.2% agarose gel on the Geldoc XR system (Bio-Rad, Hercules, CA) and analyzed by Sanger sequencing.

### Tracking of indels by decomposition (TIDE)

To evaluate the efficiency of Cas9 D10A and sgRNAs in generating indels, genomic DNAs were PCR amplified using primers F1/R1 and CloneAmp HiFi PCR premix (Takara, Mountain View, CA) or Q5 High-Fidelity 2X Master Mix (NEB). PCR product was purified and submitted for Sanger sequencing, and the sequence traces were analyzed using TIDE^14^ (http://shinyapps.datacurators.nl/tide/), a decomposition algorithm that accurately estimates indel frequency in a cell population.

### Off-target analysis

To predict potential off-target effects, guide sequence for gRNA-A was analyzed using Synthego CRISPR design tool (https://www.synthego.com/products/bioinformatics/crispr-design-tool) and the top ranked 10 hits were selected for screening. Off-target (OT) Primers (OT-F/OT-R; Table S1) were designed to amplify 400–600 bp regions spanning each potential off-target site using Q5 High-Fidelity 2X Master Mix. Single PCR product was confirmed for each primer pair. PCR products were Sanger sequenced to confirm the absence of indels at the predicted off-target sites.

### Immunofluorescence staining

Cells grown on coverslips were fixed with 4% paraformaldehyde for 15 min at room temperature. The cells were permeabilized using 0.2% Triton X-100 for 20 min at room temperature, then blocked with 5% Bovine Albumin Serum (BSA) in PBS for 1 h at room temperature. Sox2 and Oct4 primary antibodies (Cell Signaling Technologies, Danvers, MA), and fluorescent secondary antibodies were used. Images were acquired using a Zeiss inverted confocal microscope.

### G-banded karyotyping

Karyotyping and G-banding analysis was performed by WiCell cytogenetics (Madison, WI).

### Statistical analysis

Statistical analyses were performed with GraphPad Prism 8.0 (GraphPad Software, Inc., San Diego, CA).The results were analyzed using one-way analysis of variance (ANOVA) followed by Tukey’s multiple comparison. Statistical significance was set at P value < 0.05.

## Supplementary Information

Supplementary Information
