# Supplementary material for: Use of single guided Cas9 nickase to facilitate precise and efficient genome editing in human iPSCs
Source: Sci Rep. 2021 May 10;11:9865. doi: 10.1038/s41598-021-89312-2 (PMC8110799; doi:10.1038/s41598-021-89312-2)
Supplement: Supplementary file 1 — Supplementary Information [file 41598_2021_89312_MOESM1_ESM.docx]

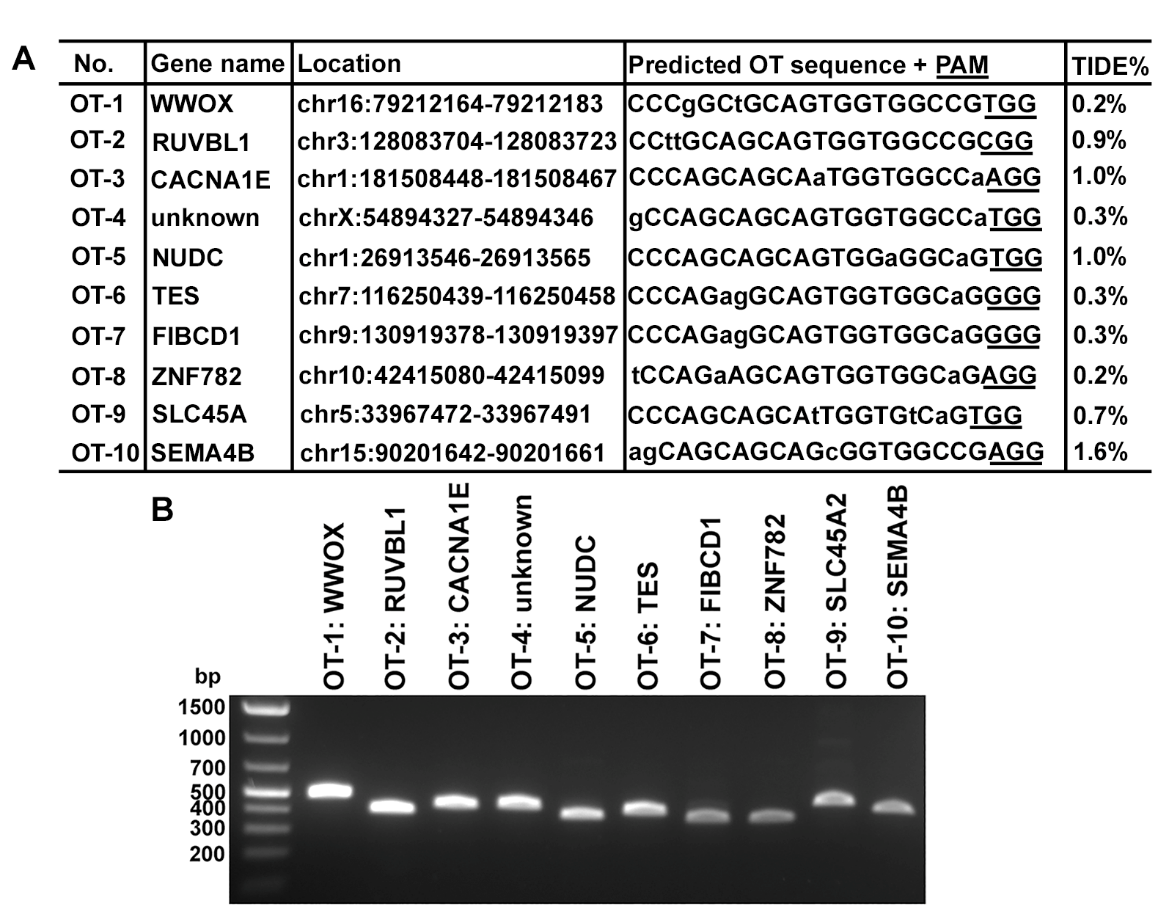


Fig. S1. SN does not generate indels in any of the top 10 predicted off-target sites. (A). A table showing the top 10 predicted off-target sites by gRNA-A. A pool of ~50 puromycin-resistant SN clones were used for PCR amplification of the 10 sites and TIDE analysis was used to determine the indel frequencies. Sanger sequencing tracks from untreated hiPSCs were used as reference. (B). PCR amplification of the top 10 predicted off-target sites showing single bands used for Sanger sequencing.


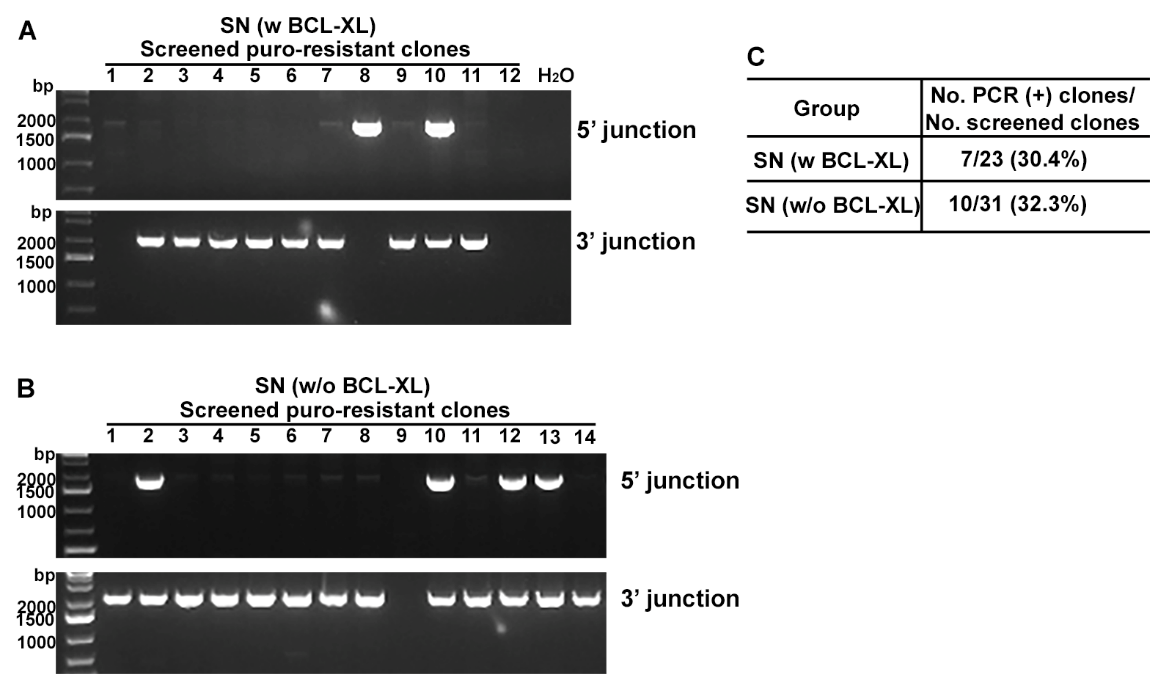


Fig. S2. Transient BCL-XL overexpression does not promote SSB-induced HDR in hiPSCs. (A-B) PCR-based screening of 5’ and 3’ junctions to identify successfully targeted iPSC clones from SN with (A) or without (B) BCL-XL overexpression. (C) Summary of targeting efficiency.


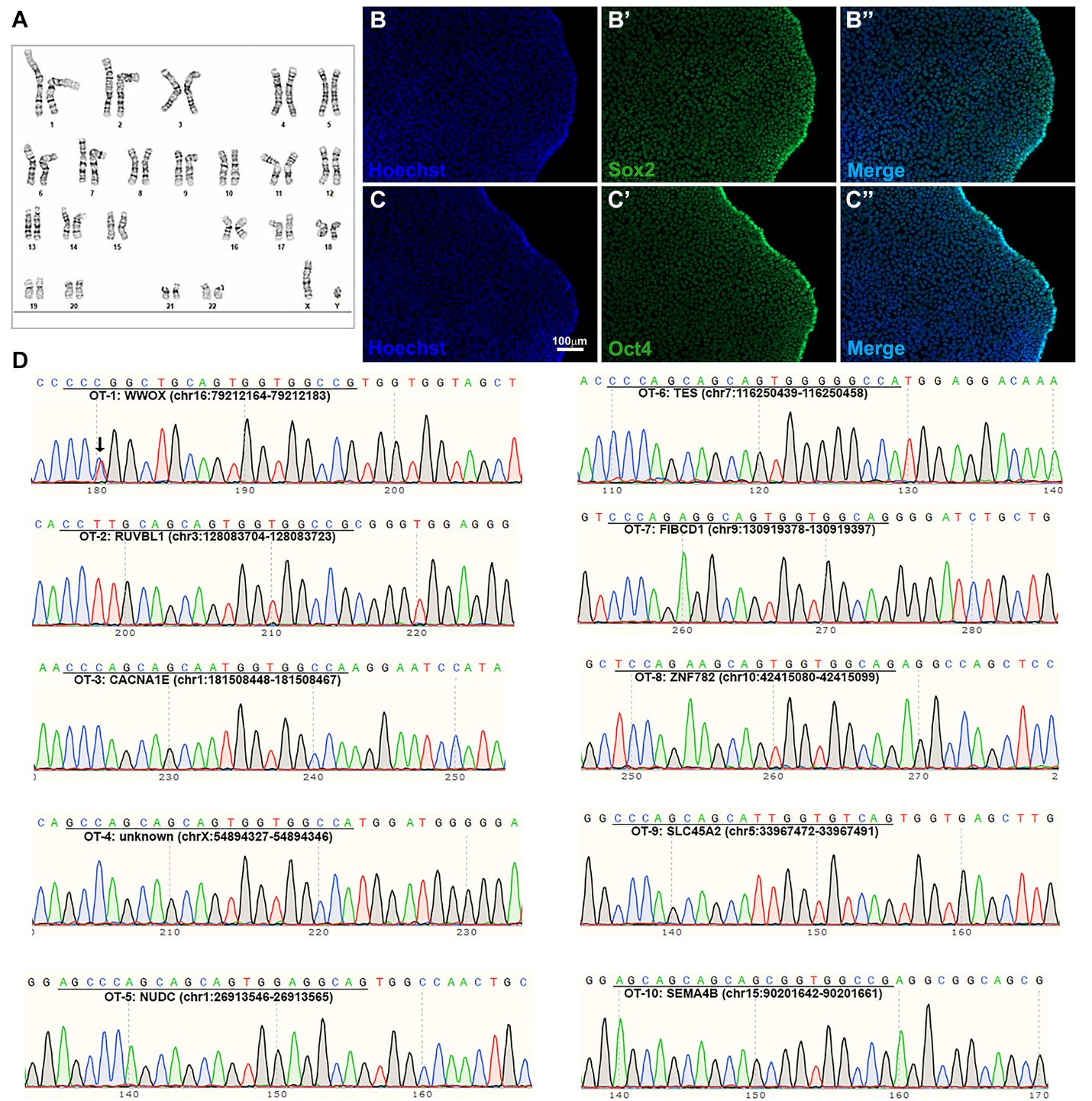


Fig. S3. Characterization of the isogenic SCA12 iPSC line generated by genome editing. (A) Normal karyotype. (B-C’’). Expression of Sox2 and Oct4 pluripotent markers in the nucleus. (D) Single nicking by Cas9n and sgRNA-A did not generate indels in the top 10 predicted off-target (OT) sites (underlined), as shown by Sanger sequencing. OT-1 site in SN-2C line had a SNP (arrow) that was also present in the unedited parental line (data not shown). Human GRCh38/hg38 was used as the reference genome.

Table S1. Primer sequences

| F1 | 5’-GTAGACGTGGCCCTTAGCTG-3’ |
| --- | --- |
| R1 | 5’-CAAAATGGTGCCTTTCTGGA-3’ |
| F2 | 5’-CGATCTGTGGAAGGGACATT-3’ |
| R2 | 5’-CCGATAAAACACATGCGTCA-3’ |
| F3 | 5’-ACTTACCGCATTGACAAGCACG-3’ |
| R3 | 5’-AGCTTGCTGAACCCTTTTGCTTT-3’ |
| F4 | 5’-CTACATACCTCGCTCTGCTAATC-3’ |
| R4 | 5’-CGAAACCCGACAGGACTATAAA-3’ |
| F5 | 5’-CACTGACTGACTGACTGGAAAG-3’ |
| R5 | 5’-GCGAGAAAGGAAGGGAAGAAA-3’ |
| PBx-F | 5’-CGACGGTACCGCGGGCCCGGGATCCACCGCCACCATGGGTAGTTCTTTA  GACGATGAG-3’ |
| PBx-R | 5’-TGATTATGATCTAGAGTCGCGGCCGCTCAGAAACAACTTTGGCACATATC  AATATTATG-3’ |
| OT-F1 | 5’-CACCTGGTAGTTCTGCGTATTC-3’ |
| OT-R1 | 5’-ACACAGATCCGCAAGAGTAAAG-3’ |
| OT-F2 | 5’-CCACCACACCCACCTAATTT-3’ |
| OT-R2 | 5’-AGGAGAGCCAGAGGTTTACT-3’ |
| OT-F3 | 5’-GCTAGCATGACCACCTTCTAA-3’ |
| OT-R3 | 5’-TGGCCTGCATCTTAGGAATAC-3’ |
| OT-F4 | 5’-GTGAGGGAAACAGTGGCTATAC-3’ |
| OT-R4 | 5’-GCTATCTGGAGACTGTGTGATG-3’ |
| OT-F5 | 5’-CTCAGGAACCTGCCATCCTTCT-3’ |
| OT-R5 | 5’-CAAACCCTTAGAAGCTACCTTCCCT-3’ |
| OT-F6 | 5’-GTGAAGGAGATGGAGCAGTTT-3’ |
| OT-R6 | 5’-GCCAATACCAACTACTGTCTGT-3’ |
| OT-F7 | 5’-CCAGTTCACATCTGCCTGTAA-3’ |
| OT-R7 | 5’-GCACGATGAACGAAGGAAATG-3’ |
| OT-F8 | 5’-GCAAAGGACATAACAGCAAATGA-3’ |
| OT-R8 | 5’-TCAACAGAGGGACAAGATAAATGT-3’ |
| OT-F9 | 5’-ACCCTATTCATATCTGCAAACTACC-3’ |
| OT-R9 | 5’-CTGACCCTATTGTGGCATGTAG-3’ |
| OT-F10 | 5’-CCTCCTTGGTCCTTTGTCAC-3’ |
| OT-R10 | 5’-TGACCCTGTTTCCCACCT-3’ |
